# Case of *Babesia crassa*–Like Infection, Slovenia, 2014

**DOI:** 10.3201/eid2605.191201

**Published:** 2020-05

**Authors:** Katja Strasek-Smrdel, Misa Korva, Emil Pal, Mojca Rajter, Miha Skvarc, Tatjana Avsic-Zupanc

**Affiliations:** Institute of Microbiology and Immunology, Faculty of Medicine, University of Slovenia, Ljubljana, Slovenia (K. Strasek-Smrdel, M. Korva, M. Skvarc, T. Avsic-Zupanc);; Murska Sobota General Hospital, Rakican, Slovenia (E. Pal);; University Medical Centre Ljubljana, Ljubljana (M. Rajter)

**Keywords:** *Babesia crassa*–like infection, *Babesia crassa*, emerging pathogen, humans, parasitemia, digital PCR, Slovenia, parasites, serology, blood smear, 18S rRNA, zoonoses, vector-borne infections, babesiosis

## Abstract

We report a case of *Babesia crassa*–like infection in an asplenic patient in Slovenia in 2014. We diagnosed the infection using microscopy, 18S rRNA sequencing, and serology and monitored parasitemia using digital PCR. With its increasing occurrence, babesiosis should be included in differential diagnoses for immunocompromised patients displaying fever.

*Babesia* infections occur worldwide and cause disease mainly in animals, but disease occurs occasionally in humans. Infections in humans are mostly attributable to *B. microti*, *B. duncani*, and *Babesia* sp. MO1 in North America; *B. divergens*, *B. venatorum*, and *B. microti* in Europe; and *B. venatorum*, *B. crassa*–like parasite, *B. microti*, *Babesia* sp. XXB/HangZhou, and *Babesia* sp. KO-1 in Asia (*1*,*2*). Transmission occurs predominantly through tick bites, but humans have acquired infections via contaminated blood products and through the transplacental and perinatal routes (*1*). Most patients with *Babesia* infections in Europe were reported to be asplenic or immunocompromised. Typical clinical signs and symptoms include fever (up to 40°C), parasitemia (20%–80%), severe anemia, muscle weakness, fatigue, and late-onset jaundice with dark urine, and sometimes complications develop. Long-term clinical follow-up that includes blood smear examination and PCR analysis is necessary because relapse and persistence of parasitemia can occur in spite of treatment. The application of novel molecular methods has revealed that the host range of many *Babesia* species is less restricted than previously thought. New species or animal pathogens are increasingly being discovered as causing *Babesia* infections in humans. We report a *B*. *crassa*–like infection in a patient in Slovenia in 2014.

 In 2014, a 55-year-old woman, living on the outskirts of Murska Sobota, Slovenia, sought medical treatment for a 6-day history of intermittent fever up to 39°C, myalgia, headache, poor appetite concomitant with weight loss, fatigue, sweating, and dark urine. She previously had a splenectomy and partial pancreatectomy 5 years previous because of cystic adenoma and adrenal incidentaloma without hormonal activity. She reported no history of travel, tick bite, animal contact, or blood transfusions.

Her blood pressure was 115/70 mm Hg, heart rate 83 beats/min, and body temperature 36.6°C, and a physical examination indicated no significant clinical findings. The first basic blood analysis revealed thrombocytopenia (platelets 85 × 10^9^/L). A differential blood analysis indicated that the concentration of large unstained cells was elevated (0.41 × 10^9^/L, reference range 0–0.4 × 10^6^/L). Biochemical laboratory testing showed mild fluctuations in liver functioning: aspartate aminotransferase 1.22 (reference range 0.17–0.51) µkat/L, alanine aminotransferase 1.13 (reference range 0.17–0.68) µkat/L, γ-glutamyltransferase 1.08 (reference range 0.03–0.51 µkat/L) µkat/L, and alkaline phosphatase 1.88 (reference range 0.5–2.0) µkat/L. C-reactive protein was 51 mg/L (150 [reference range 0.76–28.5] nmol/L), and mild erythrocyturia was present. Giemsa-stained blood smears showed unusual inclusions in erythrocytes, Howell-Jolly bodies, mild anisocytosis, some atypical lymphocytes, and some large thrombocytes. We observed many ring forms and some paired piriform shapes of *Babesia* spp. in blood smears (Figure), and parasitemia was 1% (Appendix Table). We confirmed diagnosis by conventional PCR and sequencing of the 18S rRNA gene (*3*). A phylogenetic analysis indicated the pathogen was the *B. crassa*–like parasite (Appendix Figure).

**Figure:**
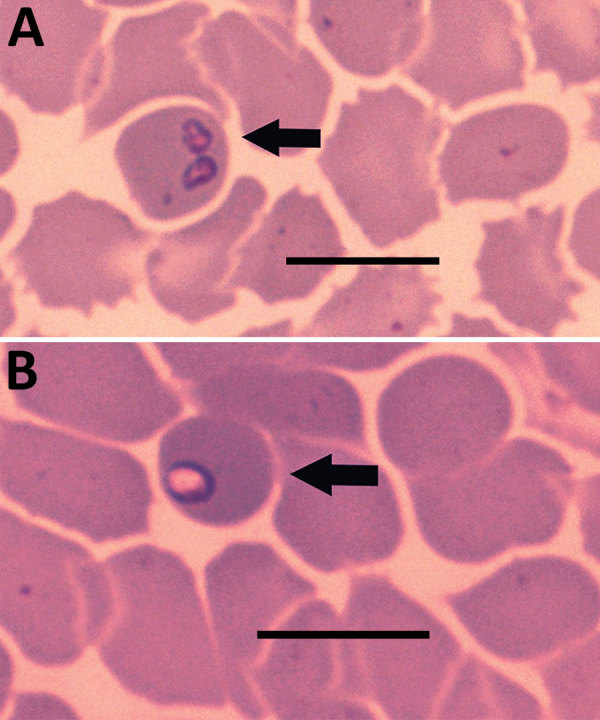
Piriform (A) and ring shapes (B) in blood smear of sample taken from patient with *Babesia crassa*–like infection, Slovenia, 2014. Smear was Wright-Giemsa stained. Scale bars indicate 50 μm.

We gave the patient an oral treatment of clindamycin (600 mg 3×/d) and quinine (600 mg 3×/d). Three days later, the patient was normothermic, and after a total of 6 days, she was discharged from the hospital with platelet levels within the reference range (150–350 × 10^9^/L). She continued the dual therapy for 14 days. To follow up on the patient’s response to treatment, we measured parasitemia levels by blood smear microscopy, PCR (*3*), and digital PCR (Appendix).

We later confirmed the infection by serology using an indirect immunofluorescence assay specific to another member of the large *Babesia* group, *B. divergens* (MegaFLUO BABESIA divergens; Megacor, https://www.megacor.at). Antibodies were cross-reactive, and results demonstrated a 4-fold increase in IgG titer (Appendix Table).

Reports of babesiosis in humans are increasing with the increase in number of immunocompromised persons; a species previously known only as an animal pathogen is posing a greater threat to those with weakened immune systems. *B. crassa* has been detected in sheep in Iran (*4*), goats and ticks in Turkey (*5*,*6*), and ticks in Hungary (*7*), and a case series of infections with *B. crassa*–like parasite in humans, sheep, and ticks was reported in northeastern China (*8*).

We report an infection of *B. crassa*–like parasite in an asplenic person in Europe that was confirmed by blood smear examination, PCR, sequencing, and serology (with assay specific to distant relative *B. divergens*). The patient recovered after treatment with the standard dual antimicrobial regimen. In addition to blood smear, we used a unique digital PCR assay to follow the decrease in concentration of babesial DNA in the patient’s blood until complete recovery. Note that DNA levels in blood do not necessarily correlate with levels of live pathogen (i.e., active infection).

With the development of new and more sensitive diagnostic techniques, parasites like *Babesia* spp., primarily recognized as animal pathogens, are becoming increasingly reported as human pathogens too, even in areas where the parasite has not been reported previously. Babesiosis should be included in the differential diagnoses for immunocompromised patients displaying fever worldwide.

Appendix More information about case of *Babesia crassa*–like infection, Slovenia, 2014.
