# Supplementary material for: Case of Babesia crassa–Like Infection, Slovenia, 2014
Source: Emerg Infect Dis. 2020 May;26(5):1038–40. doi: 10.3201/eid2605.191201 (PMC7181921; doi:10.3201/eid2605.191201)
Supplement: Appendix — More information about case of Babesia crassa–like infection, Slovenia, 2014. [file 19-1201-Techapp-s1.pdf]

Article DOI: https://doi.org/10.3201/eid2605.191201

Case of *Babesia crassa*–like Infection, Slovenia, 2014

Appendix

To follow up a patient's response to the treatment we measured the level of parasitemia with blood smear using microscopy, conventional PCR (*1*) and digital PCR (dPCR) (Appendix Table). While peripheral blood smears were positive only until 5th day of hospitalization, DNA was detected for one and a half months (Appendix Table). At the last follow-up visit, three months later, both conventional PCR, dPCR and blood smear were negative. The second day after beginning of the treatment the parasitemia started to decrease. The blood smear was negative after five days, where DNA concentration decreased for 1.6 log (Appendix Table). In the following days a consistent, but slow decrease of DNA was demonstrated until the patient became completely negative three months later (Appendix Table). By using digital PCR, we have demonstrated that novel molecular techniques are more sensitive than direct blood smear microscopy or conventional PCR.

PCRs, Sequencing and Phylogenetic Analysis of a Part of Genome of *Babesia* spp.

To investigate which species is responsible for the patient's disease we first sequenced the PCR (*2*) product (353 bp) of 18S rRNA gene of *Babesia* spp. After analysis the sequence of the product was compared with previously published sequences deposited in GenBank by BLAST. We retrieved the 99% identity with sequence of *Babesia* spp. AM-HC344 from a tick *Haemaphysalis concinna* from Russia (Far East, Amur Region; GenBank Acc. No. KJ486564). As a next step we performed a conventional PCR on a nearly complete 18S rRNA gene with primers CryptoF and CryptoR (*3*). Approximately 1700 bp long product was sequenced with additional sequencing primers (*3*). After the comparison of a sequence of the product with published sequences we retrieved 98% identity with *Babesia crassa* from sheep in Iran (Acc. No.

AY260176). The sequence of a nearly complete 18S rRNA gene of *B. crassa*-like babesia from a Slovenian patient is deposited in the GenBank under Acc. No. MK240324.

To confirm the result of a sequencing of 18S rRNA gene we performed additional conventional PCR amplifying a partial segment of a beta-tubulin gene (748 bp) of *B. crassa*–like (*1*) pathogen. After sequencing with additional sequencing primers on both strands, analysis and comparison with sequences in GeneBank we retrieved 99% identity with a sequence of *B. crassa*-like (Acc. No. KX827593) from a tick in China. The sequence of 18S rRNA gene of *B. crassa*-like babesia from a Slovenian patient is deposited in the GenBank under Acc. No. ML230987.

In a phylogenetic tree of partial 18S rRNA gene a sequence of a babesia from our patient clustered together with *B. crassa* – like sequences from ticks from China and from sheep from Iran and Turkey (Appendix Figure).

Quantification of Parasitemia with Digital PCR

For quantification of *Babesia crassa* – like pathogen we have developed a digital PCR (dPCR) on QuantStudio™ 3D Digital PCR System (Thermo Fisher Scientific, Applied Biosystems, USA). The Primer Express Software v3.0.1 (Thermo Fisher Scientific, Applied Biosystems, USA), with default conditions, was used for designing primers Bab_Irk_F1 (5'-CTA GCT GTC GAG AGA TAG TTT CGA CT-3'), Bab_Irk_R1 (5'-GCA TCA CAG ACC TGT TAT TGC CTT-3') and probe Bab_Irk_P1 (5'-6FAM-AGA GGG ACT CCT GTG CGT CAA GCG TAG GGG-BHQ1-3') targeting 94 base pair (bp) long hypervariable region of *Babesia* spp. 18S rRNA. Specificity of developed primers and probe was checked with BLAST.

For the QuantStudio™ 3D digital PCR system, the reactions were prepared in a final volume of 15 µL, composed of: 7.5 µL of Mastermix QuantStudio® 3D Digital PCR, 0.3 µL of Bab_Irk_F1 (50 µM), 0.3 µL of Bab_Irk_R1 (50 µM), 0.2 µL of Bab_Irk_P1 (20 µM), 5.2 µL of water and 1.5 µL of DNA at different dilutions. Results were expressed as DNA copies per µL. The reaction mix was loaded onto the QuantStudio 3D digital PCR chips by using QuantStudio 3D digital PCR chip loader. The amplification conditions were: 95 °C for 20 sec, cycling conditions: 95 °C for 3 sec, 58 °C for 3 sec, 60 °C for 30 sec (40 cycles). The amplifications were performed in a Proflex™ 2x Flat PCR System. The chips were transferred to the

QuantStudio 3D Instrument for imaging. Data elaboration was executed using the cloud-based QuantStudio 3D Analysis Suite software (version 3.0.03) in the absolute quantification module maintaining automatic settings. For each run, at least one negative control was included. The quality threshold was set at the default value of 0.5, to define the accepted wells and ranged from 13,265 to 21,912 with a mean of 17,136.

References

1. Jia N, Zheng YC, Jiang JF, Jiang RR, Jiang BG, Wei R, et al. Human babesiosis caused by a *Babesia crassa*-like pathogen: a case series. Clin Infect Dis. 2018;67:1110–9. PubMed http://dx.doi.org/10.1093/cid/ciy212

2. Olmeda AS, Armstrong PM, Rosenthal BM, Valladares B, del Castillo A, de Armas F, et al. A subtropical case of human babesiosis. Acta Trop. 1997;67:229–34. PubMed http://dx.doi.org/10.1016/S0001-706X(97)00045-4

3. Holman PJ, Madeley J, Craig TM, Allsopp BA, Allsopp MTEP, Petrini KR, et al. Antigenic, phenotypic and molecular characterization confirms *Babesia odocoilei* isolated from three cervids. J Wildl Dis. 2000;36:518–30. PubMed http://dx.doi.org/10.7589/0090-3558-36.3.518

Appendix Table. Results of microbiological tests of blood samples from day one to complete recovery (day 113)*

Day of visit/hospitalization	Blood smear (%)	Parasitemy – dPCR result (log DNA/ml)	IIF IgG (titer)
1st	1.0	10.8	1:32
2nd†	NA	10.7	
3rd	0.6	10.1	
4th	0.4	10.0	
5th	0.2	9.3	
6th	negative	9.5	
8th	negative	8.4	1:512
9th	negative	7.9	
13th‡	negative	7.5	
23th	negative	7.1	1:512
35th	NA	6.7	
49th	NA	6.1	1:512
90th	negative	5.0§	1:512
105th	negative	negative	
113th	negative	negative	

*dPCR, digital PCR; IIF, indirect immunofluorescence; NA, not available.
†start of treatment.
‡end of treatment after fourteen days.
§conventional PCR negative.

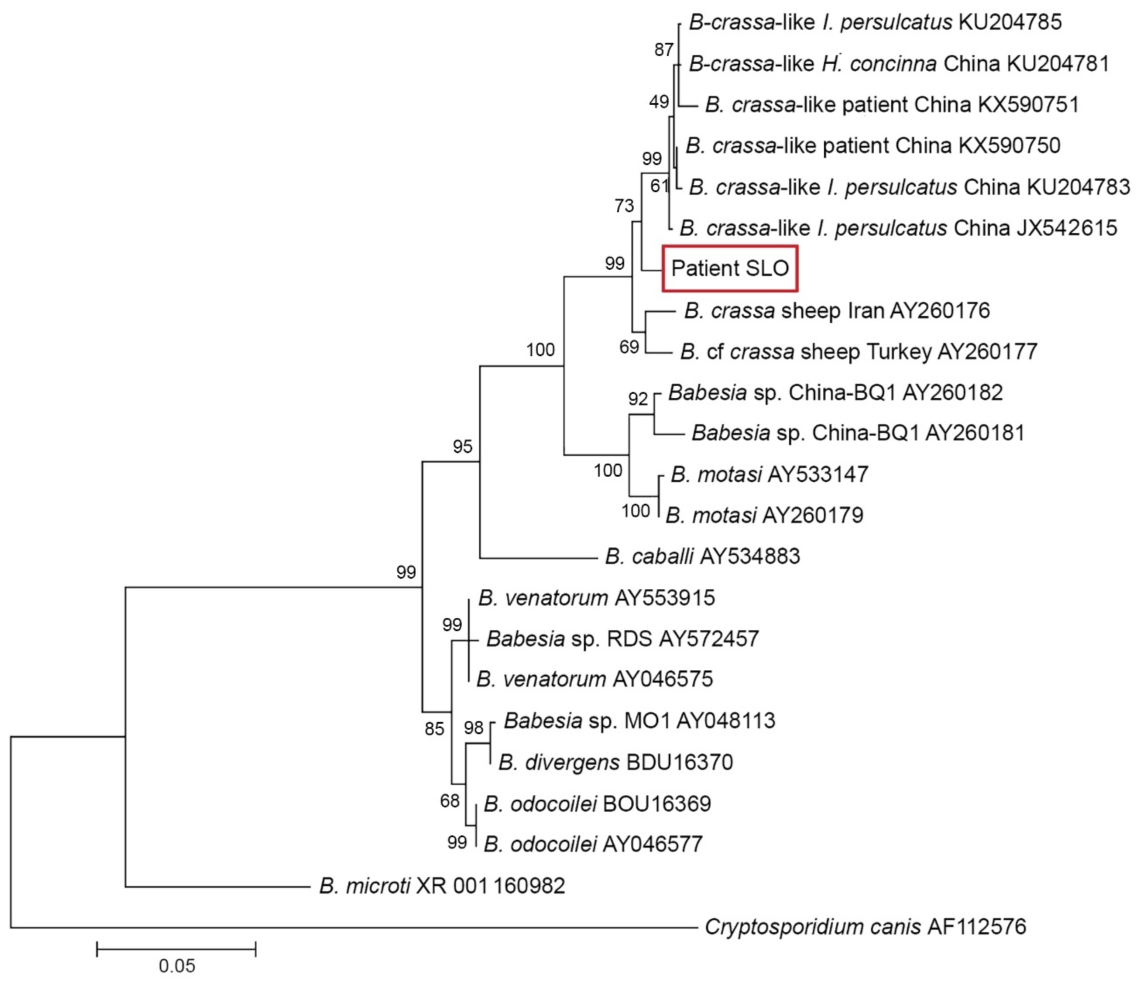

Appendix Figure. A phylogenetic tree of partial 18S rRNA gene sequences of *Babesia* spp. using Neighbour – joining method. The tree is rooted using *Cryptosporidium canis* as an outgroup. The bootstrap values based on 1000 replicates are displayed next to the branches. Sequences of babesiae and *Cryptosporidium sp.* and corresponding accession numbers were retrieved from GeneBank on January 2019. Accession number of partial 18S rRNA gene sequence of *Babesia* spp. from Slovenian patient is MK240324. SLO, Slovenia.
